# Target selection for CAR-T therapy

**DOI:** 10.1186/s13045-019-0758-x

**Published:** 2019-06-20

**Authors:** Jianshu Wei, Xiao Han, Jian Bo, Weidong Han

**Affiliations:** 1 Molecular & Immunological Department, Bio-therapeutic Department, Chinese PLA General Hospital, No. 28 Fuxing Road, Beijing, 100853 China; 2 Department of Hematology, Chinese PLA General Hospital, Beijing, 100853 China

**Keywords:** Chimeric antigen receptor-modified T cells, Coverage, Specificity, Off-tumor effect, Expression stability, Target combination

## Abstract

Chimeric antigen receptor-modified T (CAR-T) cells have achieved significant success in the treatment of several hematological malignancies. However, the translation of the existing achievements into the treatment of other tumors, especially solid tumors, is not smooth. In addition to the optimization of CAR structures, preparation, and clinical protocols, rational selecting and utilizing the targets was more pivotal. In this review, the criteria for target selection and some new strategies for targets utilization were summarized and discussed. This systematic review will help researchers better understand how the efficacy and safety of CAR-T treatment would be affected by targets and thus more rationally select targets and conduct clinical trials.

## Introduction

With the elucidation of mechanisms of tumor development, different approaches have been exploited to fight against cancers, such as chemotherapy, radiation therapy, and kinase inhibitors. Despite great advances achieved, complete remission, especially durable remission, for unresectable malignancies remains rare. To improve cancer treatments, high level of enthusiasm is always devoted to the research on novel cancer therapies, including immunotherapy.

Owing to the robust and long-lasting antitumor functions, chimeric antigen receptor modified-T (CAR-T) cells achieved significant success [1, 2]. The recognition and clearance of tumor cells by CAR-T cells are dependent on chimeric antigen receptor (CAR) molecule but not the binding of traditional T cell receptor (TCR) and human leukocyte antigen (HLA), so that the immune escape caused by low expression of HLA in tumor cells could be overcomed. CAR-T cells can differentiate into memory T cells, by which a long-term antitumor activity can be established. Due to these advantages, CAR-T cells exhibit potent antitumor activity in the treatment of hematological tumors.

The complete remission rate (CRR) of CD19-targeted CAR-T (CAR-T-19) cells in the treatment of B cell acute lymphocytic leukemia (B-ALL) could be more than 90% [2, 3]. Outstanding antitumor efficiency in other hematologic malignancies such as multiple myeloma (MM) [4–7] and B cell lymphoma have also been achieved [8–11]. In 2017, the US Food and Drug Administration approved the drug CTL019 (tisagenlecleucel-T, Novartis) for the treatment of B-ALL, which was viewed as a milestone of immunotherapy.

As an emerging tumor treatment strategy, CAR-T therapy still needs further exploration to expand its clinical application, and further optimization is also needed to solve real-life clinical problems. After 20 years of exploration, we have basically understood the main factors that determine the function of CAR molecule, such as co-stimulatory molecules [9] and extra-membrane spacer regions [12]. These research results have transformed into a variety of CAR structures. Generally speaking, the second generation CAR structure containing one single co-stimulatory molecule has shown sufficient antitumor function and safety, and is the main structure used in clinic at present [13]. At the same time, advances in the preparation of CAR-T products [14–16], the protocols for clinical implementation [17–19], and the management of side effects [20, 21] have greatly improved the clinical efficacy and application scenarios.

Given that the CAR-T therapy has possessed with mature CAR structure, preparation, and clinical protocols, how to choose and utilize the target becomes the key to determine its potential.

There are no uniform guiding criteria for target selection, and the criteria are often needed to be modified according to the actual clinical needs. For example, the most urgent need for solid tumors treatment is to improve efficiency at present; therefore, choosing a target with high specificity and high coverage is of greatest value. However, this has not been a significant hurdle for the treatment of ALL and B lymphoma right now, because the CD19 or CD20 has been validated to be of sufficient coverage and specificity. Instead, the most urgent need is to further improve CRR and prevent recurrence [22, 23].

Target selection is a very important determinant, which requires researchers to implement a comprehensive assessment. But at present, there are few reviews which detailed and discussed this issue. Therefore, in this paper, we will review and discuss the principles for target selection and the new strategies for target utilization. We believe this will help the scientists to better design and implement CAR-T therapies.

## Coverage and specificity

CAR molecule targets tumor cells’ surface antigens. Not only proteins but also carbohydrate and glycolipid molecules could be the potential targets. The interaction between CAR and targets leads to the formation of immune synapses, with which the contact-dependent cytotoxicity occurs. To achieve prominent tumor clearance, CAR-T cells should target the vast majority of tumor cells, that is, the selected target antigen should have sufficient coverage on the tumor cells. At present, most of the CART therapies with good clinical effect meet the selection criteria of high coverage, such as CD19, CD20, and B cell maturation antigen (BCMA) [24]. In addition, there are some other targets with high coverage that deserve further verification, such as C-type lectin-like molecule-1 (CLL-1) for acute myeloid leukemia blasts [25].The specificity of selected targets should be good enough to prevent CAR-T cells from causing serious organ damage. There are two main toxic side effects in the CAR-T treatment, one is cytokine release syndrome (CRS) caused by activated immune cells and the other one is “off-tumor” effect caused by damage on non-tumor cells. Compared with CRS, which could be effectively managed, the “off-tumor” effect which can cause serious organ damage or even death [26] is difficult to distinguish from the antitumor effect.

Therefore, an ideal target should be of high coverage and high specificity to guarantee both effectiveness and safety. However, the “ideal” target is almost non-existent in reality.

**Fig. 1:**
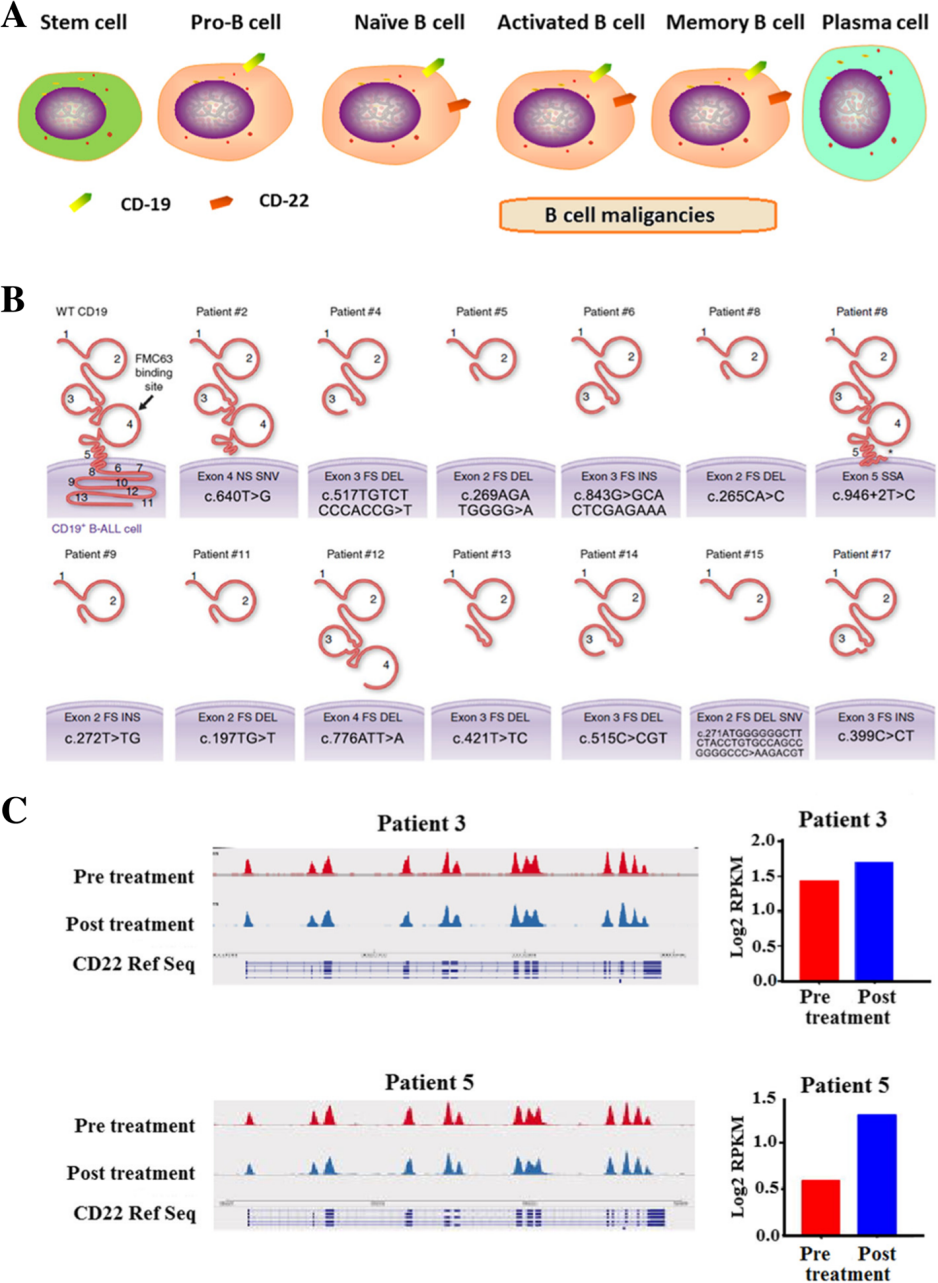
Schematic description of the expression of CD19 and CD22. **a** The expression patterns of CD19 and CD22 during B cell development. **b** Gene mutation is the main mechanism of antigen-negative relapse after CAR-T-19 treatment. This picture is quoted from an article published in *Nature Medicine* by Orlando et al. in 2018 [27]. **c** Silence of expression, rather than gene mutation, is the main cause of CD22 loss after CAR-T treatment. This picture is quoted from an article published in *Nature Medicine* by Fry et al. in 2018 [28]

Take CD19 as an example, the most widely used target in CAR-T therapy, which has been validated to be effective and safe to treat B-ALL, chronic lymphocytic leukemia (CLL), and B cell lymphoma. CD19 is widely and confinedly expressed throughout the entire phase of B cell development until terminal differentiation into plasma cells (Fig. 1a). Therefore, CD19 has perfect coverage for B cell malignancies, which made CAR-T-19 treatment achieved very high CRR.

However, in terms of specificity, CD19 is not an ideal target. Along with the antitumor effect of CAR-T-19, normal B cells will also be cleared, which leads to long-term B cell aplasia. Fortunately, B cell aplasia could be tolerated by patients due to effective clinical management. The same is true for CD20 and BCMA. Although they are not strictly expressed on tumor cells, these CAR-T treatments have shown outstanding clinical prospects due to high coverage and tolerable off-target effects.

Therefore, we insist that the coverage for single-target CAR-T treatment should be high enough. For its specificity, the off-tumor effect needs to be evaluated and tested strictly. In the implementation, the treatment intensity which needs to be accordingly set within a proper window is determined by toxic side effects. When the off-tumor side effects can be tolerated, specificity could be compromised in practice (Table 1). The experience on coverage and specificity may not be suitable for solid tumors due to the inherent heterogeneity. For solid tumors, we can hardly get a target of which the coverage is good enough. Moreover, most of the targets tested currently would bring significant off-target effects, so the treatment intensity is often limited, which in turn further weakens the effectiveness. Therefore, the treatment of solid tumors might require combination of multiple targets and endogenous antitumor effects, which will be discussed later.

**Table 1:** Determinant of target selection for CAR-T therapy

Determinant	Main effect	Selection criteria
Coverage	Tumor clearance efficiency	1. It should be high enough, and it is possible to obtain CRR response using antibodies or CAR-T therapy against this target. 2. Refer to the efficacy of related antibody drugs. 3. Test patients’ samples before the treatment.
Specificity	“Off-tumor” cytotoxicity	1. It is limited to tumor tissue, or the damage caused by off-tumor toxicity can be tolerated or well managed. 2. Refer to the side effect of related antibody drugs.
Stability	Response duration and recurrence	1. The expression should be stable and not easily regulated by external signals. 2. It plays an important role in the growth and survival of cancer cells.

## Stability

Coverage and specificity are the basic factors in target screening for CAR-T therapy. In addition, the expression stability of antigens is also fundamental.

With high evolutionary potential brought about by genomic instability, cancer cells could rapidly acquire the phenotypes that prevent immune killing. In CAR-T treatment, losing targets is a very common mechanism for the failure of treatment. Both theory and experience have proved that the less stable the target is, the easier it is for cancer cells to escape from the killing of CAR-T cells. Therefore, as an ideal target, its expression should be fixed. If the expression is floating, the treatment is hard to be effective.

Take CD19 and CD22 as an example, their expression patterns are almost the same (Fig. 1a). From the perspective of coverage and specificity, it is inferred that CAR-T-19 and CAR-T-22 therapy should present similar antitumor potential in the treatment of B cell lymphoma. However, in clinical practice, CAR-T-19 therapy exhibited more significant and persistent antitumor activity [22, 28, 29].

In patients who relapsed after treatment with CAR-T-19 or CAR-T-22, target loss is the most common cause, except for the poor proliferation and persistence of CAR-T cells. One study demonstrated that CD19 gene mutation is the main cause of target loss during CAR-T-19 treatment [27]. Among them, CD19 was not completely silenced but existed in different truncated forms (Fig. 1b). On the contrary, the CD22 was more prone to be silenced by upstream regulations [28], such as signaling pathways or epigenetic modification, rather than gene mutation (Fig. 1c). In general, signaling or epigenetic regulation occurs faster and more often than genomic mutations. In other words, the expression of CD19 is more fixed than that of CD22. We believe that this was the main reason why the clinical response of CAR-T-22 is inferior to that of CAR-T-19. Recently, a report on the substitution of scFV with FMS-like tyrosine kinase-3 ligand [30] provided a novel strategy for overcoming the problem of targeted motif loss caused by gene mutation. In addition to the regulation mechanisms, another reason for the fixed expression of CD19 is that it plays an important role in the survival and proliferation of B cell malignancies. As we know, CD19 is essential to the function of BCR complex, which is necessary for B cell differentiation and survival [31]. B cell malignancies that completely lose CD19 cannot obtain sustained dominant growth, and this also explains why the relapsed B cell malignancies after CAR-T-19 treatment were prone to express truncated CD19 that lost the scFv targeting segment rather than silencing the expression.

In conclusion, the expression of a good target for CAR-T therapy should be fixed, which is determined by its own regulation mechanisms and the importance in maintaining the proliferation of malignant cells (Table 1).

## Strategies for improving coverage and specificity

Due to the lack of ideal target, the potential of CAR-T therapy has not been fully realized in many cases [32]. Researchers have designed many novel strategies to improve the coverage and specificity of CAR-T targets.

The most common strategy is to combine different targets.

**Fig. 2:**
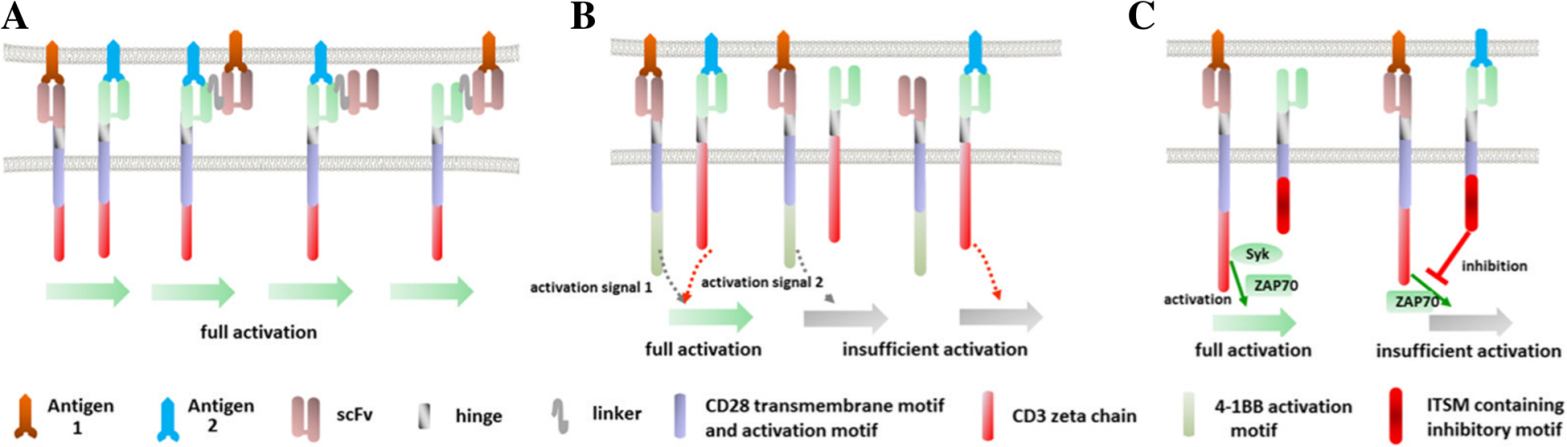
Strategies of combining two targets to improve coverage and specificity. **a** CAR-T cells can be fully activated by antigen-1 or antigen-2 to improve tumor coverage. **b** CAR-T cells can only be fully activated when antigen-1 and antigen-2 are engaged simultaneously. **c** CAR-T cells will be inhibited when antigen-2 is present

The first kind of relationship between different targets is 1 or 2 (Fig. 2a). In this approach, two intact CARs could be constructed into one vector [33]. The transfected T cells will express two CARs specific for two different antigens. Full activation could be achieved when each antigen is engaged. When the two antigens are encountered at the same time, the immune response can be further enhanced. This strategy can be generally considered as co-administration of two different normal CAR-T cells. Except for expressing two different CAR molecules, tandem expression of two scFv domains in one CAR molecule can also come up to a similar effect [34–37]. According to several reports, the relative positional changes between the two scFv domains have an important effect on the function of the CAR molecule [35, 36]. Therefore, the design of such tandem CAR may require more detailed optimization.

Extended antigen coverage could guarantee more thorough tumor cell recognition and clearance. For different tumors, the purposes of this multi-target combination strategy are different. For example, a very high CRR for ALL treatment by CAR-T-19 has been achieved, and simultaneous targeting of CD19/CD22 or CD19/CD123 may not significantly improve the initial clinical benefits. However, its main purpose is to prevent CD19-negative recurrence after CAR-T treatment. For most solid tumors, the heterogeneity is very high. Both theory and practice have proved that the efficiency of single-targeted CAR-T therapy was very limited. Therefore, the main purpose of adding a target, such as CAR-T-HER2/IL13Ra2, is to improve the coverage so that more tumor cells can be cleared, which would lead to improved response to CAR-T treatment.

It should be noted that although the 1 or 2 strategy could increase the coverage, it may also increase on-target/off-tumor risks. Therefore, more careful evaluation is required before clinical implementation.

The second kind of relationship between different targets is 1 and 2 (Fig. 2b). In this strategy, the intracellular activating regions of a complete CAR molecule, the CD3ζ and co-stimulatory activation domains, are expressed separately within two half-baked CARs. The CAR-1 provides a CD3ζ-mediated activation signal upon recognition of antigen 1 (just like the 1st generation CAR), and the co-stimulatory signal is provided by the CAR-2 when antigen 2 is engaged. In 2013, Kloss et al. at MSKCC demonstrated that recognition of two targets was necessary for full activation in this design [38]. In that article, the half-baked CAR molecule which provided co-stimulatory signals was called chimeric co-stimulatory receptor.

In this manner, the combinatorial CAR-T cells can become fully activated only when they met with 1^positive^/2^positive^ target cells. And this combinatorial activation system is considered to hold promising selectivity for solid tumors.

However, it is worrying that the activated T cells might recirculate and encounter 1^positive^/2^negative^ or 1^negative^/2^positive^ target cells. Without requiring for full activation, the CAR-Ts might clear these target cells, thus resulting in an “on-target off-tumor” effect.

The third kind of relationship between the two targets is 1 not 2 (Fig. 2c). In this concept, the CAR-Ts’ activation or function would be inhibited by an inhibitory CAR (iCAR) upon expected antigen engagement. In 2013, Fedorov et al. at MSKCC developed such a kind of iCAR molecule [39]. The iCARs were designed to be consisting of scFv domain, hinge, and transmembrane domain just as the common CAR molecule. But the intracellular domain was replaced with the signaling domains of cytotoxic T lymphocyte-associated protein-4 (CTLA-4) or programmed cell death-1 (PD-1). The synthetic receptors were hypothesized to own inhibitory function which was proved to be so subsequently. From a certain point of view, iCAR can also be seen as mimetic PD-1 or CTLA-4.

After a series of verification, the iCARs were proved to be able to selectively limit T cell activation induced by endogenous TCR and exogenous CAR molecule. The antigens expressed in normal tissues but not tumor cells could be used as the iCAR’s targets. But the inhibitory effect was temporary and reversible, thus enabling CAR-T cells to function with the common activating CAR molecule. In this manner, the damage to normal tissues could be diverted without eliminating or irrevocably inhibiting the CAR-T cells.

## Extending the targets

The neoantigen derived from gene mutation is an ideal target because it can fully distinguish cancer cells from normal ones. Most of the identified neoantigens are intracellularly expressed and can only be immunogenic in the form of peptide/HLA complex. Therefore, it is generally not considered to be an applicable target for CAR-T treatment.

The concept of using scFv targeting epitope/HLA complex to construct CAR makes it possible for intracellular proteins to be the candidate targets. One example is Wilms’ tumor-1(WT-1) [40], which is an oncogenic transcription factor overexpressed in many malignancies. The researchers obtained WT-1/HLA-A*02:01 complex-specific scFv by phage display technology [41], which was then constructed into traditional CAR molecule. The HLA complex targeted CAR-T cells were then confirmed to be effective to kill tumor cells specifically. The greatest advantage of this strategy is its high specificity, so the potential off-tumor effect can be limited to a very low level. However, it could be anticipated that the stability and coverage of this kind of target might not be good enough. Therefore, in the future clinical application, we believe that the combination use of the peptide/HLA targets is needed. In addition, a fundamental question needs to be carefully studied before this strategy is widely implemented, that is, whether its specificity is really as good as it is supposed to be.

**Fig. 3:**
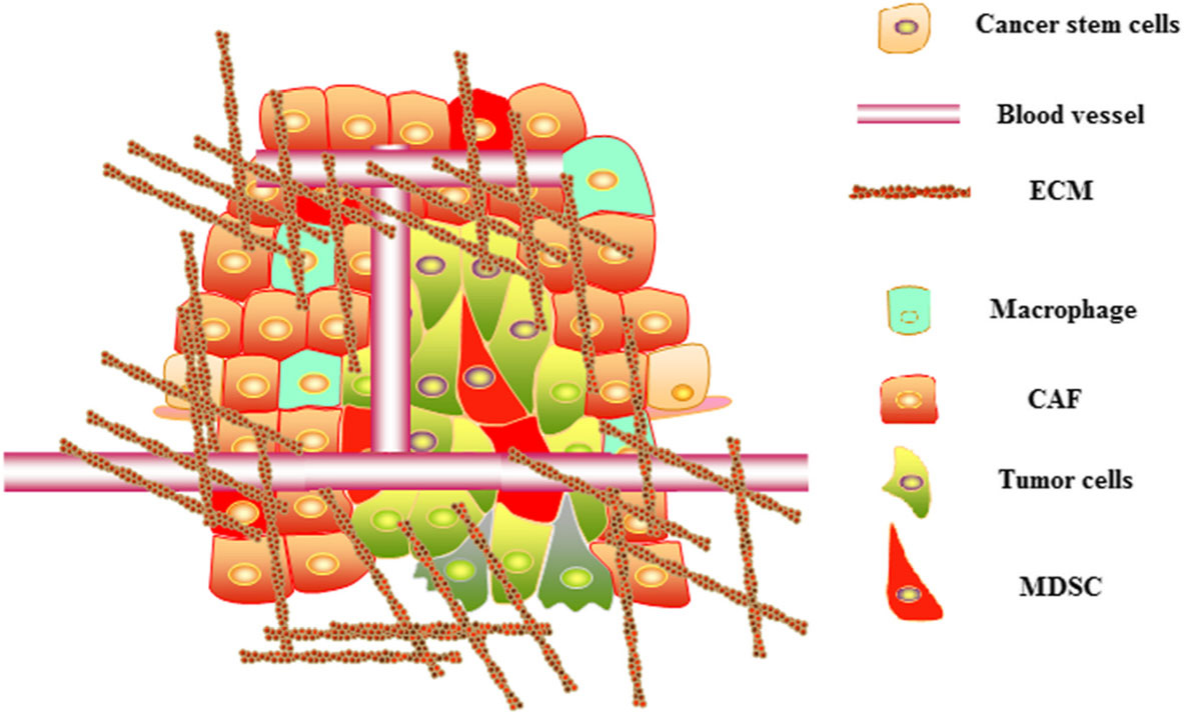
Other cells that play an important supporting role in the growth of tumors can also be targeted for CAR-T therapy

Solid tumors establish a sophisticated composition to support tumor growth, including immunosuppressive microenvironment, unique vascular system, and nutritional environment suitable for tumor growth and so on. The cells involved in the establishment and maintenance of the microenvironment can also be the targets for CAR-T therapy (Fig. 3). For example, the cancer-associated fibroblasts (CAF), which support tumor growth by secreting growth factors, chemokines, and extracellular matrix, could be destroyed by CAR-T cells targeting fibroblast activation protein (FAP) [42], and potent antitumor effects by CAR-T-FAP have been also confirmed [43, 44]. In addition, destroying tumor vascular system [45, 46] and killing cancer stem cells [47, 48] by CAR-T cells have also been proved to be feasible and effective. The strategy that transforms the targets from cancer cells to other cells supporting the growth of tumors also provides a basis for combined application of other treatments.

The CAR-T cells we made generally possess unitary specificity, which means only one target could be recognized. This limits the applicability of CAR-T cells, especially for the treatment of highly heterogeneous solid tumors. It will be meaningful to reform CARs to be specific to wider range of antigens. As early as 2012, the researchers at the University of Pennsylvania (UP) synthesized a novel CAR structure [49], of which the specificity was variable. In this article, the researchers replaced the scFv domain with a modified avidin motif linked to the rest part of CAR molecule. The T cells could recognize different targets with the help of different biotinylated molecules, such as biotinylated tumor-specific antibodies and ligands. The versatility afforded by the novel CAR structure made it feasible for sequential or multiple-target CAR-T therapies achieved within one treatment. Subsequently, there are several similar work published, in which anti-PNE (peptide neo-epitope) scFV [50], leucine zipper [51], anti-5B9-tag scFV [52], and anti-FITC scFV [53] were used to construct the universal CAR.

In this manner, a specific molecule, normally antibodies, works as an adaptor between T cells and tumor cells. Except for controllable specificity, the activity of CARTs activity could also be precisely controlled with titration or removal of the adaptors.

## Triggering endogenous immunity

In the treatment of solid tumors, what roles CAR-T cells should play in addition lysis tumor cells is worth further exploration.

There are currently many strategies to improve the accuracy of CAR-T therapy in the treatment of solid tumors, but solid tumors are so complicated (especially with very high heterogeneity) that the CAR-T cells which target a specific target can hardly cover all solid tumor cells, even if multiple targets could be combined. From a certain point of view, CAR-T cells seem to be naturally inadequate for overcoming the heterogeneity obstacle.

Due to the extremely high abundance of TCR in vivo, compared to the CAR-T cells recognizing a specific target, the endogenous tumor-specific T cells (recognizing neoantigen and HLA complexes by TCR) are more likely to fully cover solid tumor cells and thus may eliminate the malignancies more clearly [54, 55]. At present, this view has been accepted by many scientists, and some meaningful exploration has been made [56].

**Fig. 4:**
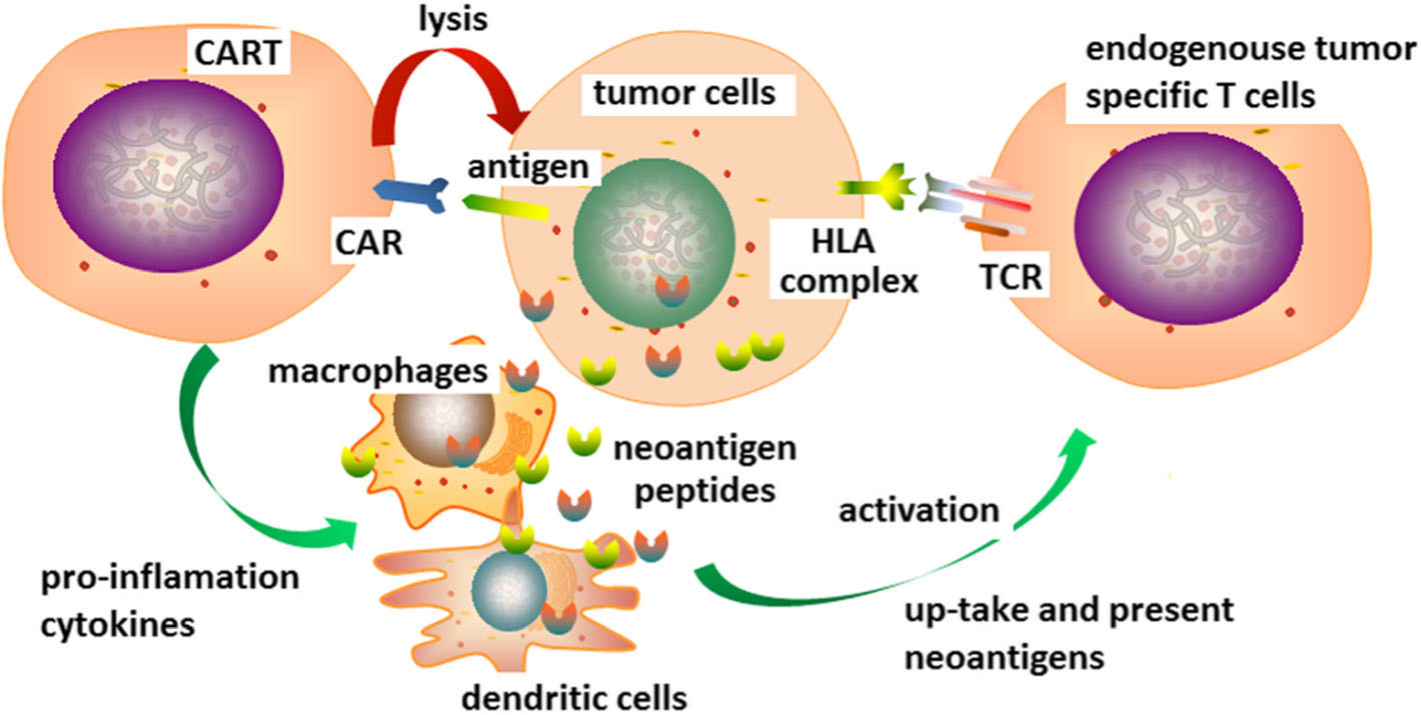
CAR-T cells can be modified to initiate and boost the endogenous tumor-specific immune response

In this scenario, CAR-T cells could be used as therapeutic tools to activate the antitumor activity of endogenous immune system. Many clinical cases have confirmed that lymphocyte infiltration in solid tumors would increase after CAR-T treatment. In addition to CAR-T cells themselves, the infiltration of endogenous dendritic cells (DCs), macrophages, and endogenous T cells could also be increased. In the activation loop (Fig. 4), the neoantigens released after CAR-T cells attacking could activate the more specific endogenous tumor-specific immune response if they are uptaken and presented by antigen-presenting cells. In addition, the CAR-T cells could be modified to release pro-inflammatory factors and form a favorable microenvironment for inflammatory response in the local area of tumors, which would further boost the endogenous tumor immune response. Under this conception, the choice of target in solid tumors CAR-T treatment does not necessarily follow the principles discussed previously. For example, the coverage does not need to be very high, as long as it can ensure that a significant immune response can be triggered.

## Switching the original signals

In addition to the heterogeneity mentioned above, the immunosuppressive microenvironment is also an important factor limiting the effectiveness of immunotherapy for solid tumors. Among the factors that establish immunosuppression, the PD-1/PD-L1 axis plays a major role. As we know, the PD-1 receptor could strongly inhibit T cell activation and proliferation upon the interaction with its ligands PD-L1 or PD-L2. The expression of PD-L1 is closely upregulated by some cytokines (especially IFN-γ), of which the release is an inherent event of immune response. Therefore, when CAR-T cells attack solid tumor cells, such a negative feedback regulation is generally inevitable.

**Fig. 5:**
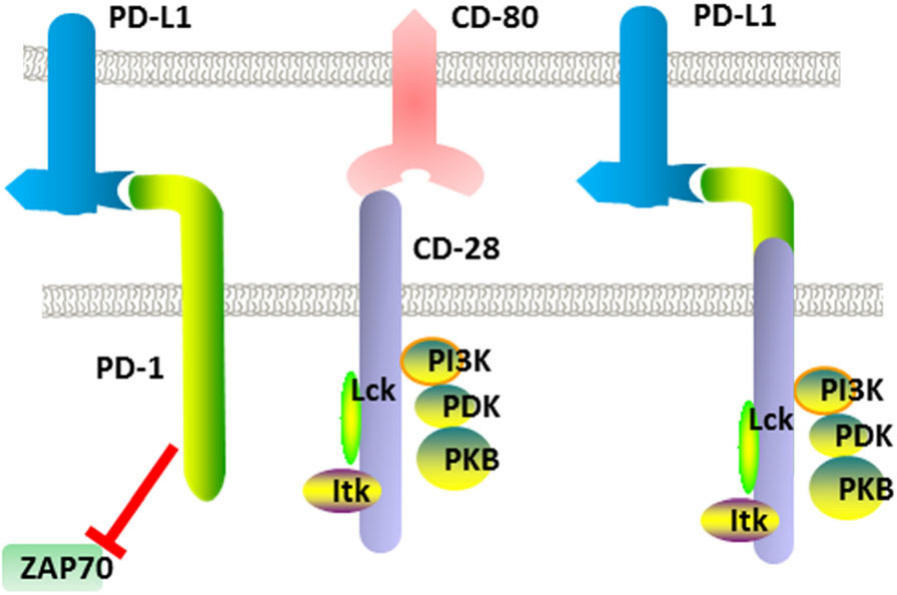
Chimeric receptors can convert inhibitory signals into activating ones

To overcome this dilemma, a PD-1-CD28 fusion receptor was firstly designed in 2012 by Prosser et al. at the City of Hope National Medical Center [57], and this chimeric receptor was expected to switch original inhibitory signals into activation signals. This structure was designed because PD-1 and CD28 both belong to the CD28 superfamily, and the compatibility of function mechanisms between them had been demonstrated earlier. The authors fused the extracellular part of PD-1 with the intracellular part of CD28. When the extracellular part engaged with PD-L1, an activation signal was transmitted instead of inhibitory signal with CD28 cytoplasmic domain (Fig. 5). Along with activation via CAR, the switch receptor could enhance cytokine release, proliferation, and cytotoxicity of CAR-T cells.

In 2015, a Germany research group optimized this structure, in which the CD28 transmembrane domain was replaced by the analog of PD-1 [58]. The authors demonstrated that the YMNM motif of the CD28 was required for optimal cytokine secretion and the PYAP motif was essential for both cytokine production and proliferation.

In 2016, Liu et al. from Carl June’s lab demonstrated that this switch receptor was able to augment the cytotoxicity of CAR-Ts to large established solid tumors [59]. And the experiment date showed that the PD1-CD28 receptor was superior in controlling tumor burden compared with PD-1 blocking antibody.

In addition, blocking PD-1 signal can effectively reverse the exhaustion of CAR-T cells [60].

Following a similar design concept, CTLA-4, lymphocyte activation gene-3(LAG-3), and mucin-domain containing-3 (Tim-3) based switch receptors could also be synthesized and tested. Therefore, by switching the inhibitory signals, these immunosuppressive molecules can also be the targets of CAR-T cells to boost their antitumor functions.

## Conclusions

Target selection is the most fundamental factor determining the potential of CAR-T therapy, and its selection criteria are not monotonous. In this review, we summarized and discussed what constituted an ideal target based on existing clinical data. We believe that coverage is the primary factor to be considered, which directly determines the ceiling of CAR-T therapy. Specificity is also a basic factor to be considered. It can affect the effectiveness of CAR-T treatment by influencing the treatment intensity. In addition, the expression of an ideal target must be fixed. Otherwise, rapid and frequent target loss will lead to the failure of CAR-T treatment.

For the treatment of solid tumors, it is difficult to get the ideal target like CD19. Therefore, the role of CAR-T therapy should not be limited to killing cancer cells directly, such as activating endogenous tumor immune response and destroying the growth environment of tumors. In addition, some new target utilization strategies might be meaningful to solve specific problems, such as combinatorial utilization of multiple targets and switching inhibitory signals.

## Data Availability

All data generated or analyzed in this study are included in this article. Other data that are relevant to this article are available from the corresponding author upon reasonable request.

## Abbreviations

B-ALL: B cell acute lymphocytic leukemia
CAR-T: Chimeric antigen receptor-modified T
CRR: Complete remission rate
CRS: Cytokine release syndrome
CTLA-4: Cytotoxic T lymphocyte-associated protein-4
DCs: Dendritic cells
HLA: Human leukocyte antigen
LAG-3: Lymphocyte activation gene-3
MM: Multiple myeloma
PD-1: Programmed death-1
PD-L1: Programmed death-ligand 1
PD-L2: Programmed death-ligand 2
TCR: T Cell receptor
TIM-3: Mucin-domain containing-3
WT-1: Wilms’ tumor-1
